# The effects of nano-silver loaded zirconium phosphate on antibacterial properties, mechanical properties and biosafety of room temperature curing PMMA materials

**DOI:** 10.3389/fcimb.2023.1325103

**Published:** 2023-12-20

**Authors:** Xingjian Chen, Tongtong Yan, Shiqun Sun, Aoke Li, Xiaorong Wang

**Affiliations:** ^1^ Jilin Provincial Key Laboratory of Tooth Development and Bone Remodeling, Department of Prosthodontics, Hospital of Stomatology, Jilin University, Changchun, Jilin, China; ^2^ Department of Prosthodontics, Hospital of Stomatology, Jilin University, Changchun, Jilin, China

**Keywords:** PMMA, AgNP Ag nanoparticle, mechanical properties, antibacterial properties, biosafety

## Abstract

Polymethyl methacrylate (PMMA) frequently features in dental restorative materials due to its favorable properties. However, its surface exhibits a propensity for bacterial colonization, and the material can fracture under masticatory pressure. This study incorporated commercially available RHA-1F-II nano-silver loaded zirconium phosphate (Ag-ZrP) into room-temperature cured PMMA at varying mass fractions. Various methods were employed to characterize Ag-ZrP. Subsequently, an examination of the effects of Ag-ZrP on the antimicrobial properties, biosafety, and mechanical properties of PMMA materials was conducted. The results indicated that the antibacterial rate against Streptococcus mutans was enhanced at Ag-ZrP additions of 0%wt, 0.5%wt, 1.0%wt, 1.5%wt, 2.0%wt, 2.5%wt, and 3.0%wt, achieving respective rates of 53.53%, 67.08%, 83.23%, 93.38%, 95.85%, and 98.00%. Similarly, the antibacterial rate against Escherichia coli registered at 31.62%, 50.14%, 64.00%, 75.09%, 86.30%, 92.98%. When Ag-ZrP was introduced at amounts ranging from 1.0% to 1.5%, PMMA materials exhibited peak mechanical properties. However, mechanical strength diminished beyond additions of 2.5%wt to 3.0%wt, relative to the 0%wt group, while PMMA demonstrated no notable cytotoxicity below a 3.0%wt dosage. Thus, it is inferred that optimal antimicrobial and mechanical properties of PMMA materials are achieved with nano-Ag-ZrP (RHA-1F-II) additions of 1.5%wt to 2.0%wt, without eliciting cytotoxicity.

## 1 Introduction

Polymethyl methacrylate (PMMA), recognized for its commendable biological, physical, and chemical properties along with superior machinability, has ascended as a predominant material in the field of stomatology (Rao et al., 2022). Employed as a material for denture reconstruction, orthodontic mobile appliances, periodontal splints, and jaw cystoccluders, room-temperature curing PMMA, while boasting numerous advantages, also manifests conspicuous disadvantages. Primarily, the rough surface and porosity of PMMA materials facilitate the colonization of microorganisms, such as Candida albicans, Streptococcus sanguineus, and Streptococcus mutans, thereby propelling the incidence of various oral diseases (An et al., 2022; Moreno-Maldonado et al., 2012; Quassem and Mahross, 2018; Sahm et al., 2022; Pourhajibagher et al., 2023; Schubert et al., 2019; Al-Dwairi et al., 2019; Patil et al., 2020; Dantas et al., 2016). Moreover, PMMA material exhibits a challenge regarding mechanical strength, proving susceptible to breakage under masticatory pressures ( Zafar, 2020; Ali Sabri et al., 2021; Alkordy and Alsaadi, 2014; Mohd Farid et al., 2022; Khan et al., 2022; Oleiwi et al., 2019; Zafar, 2018; Rana et al., 2021). Considering the myriad and multifaceted bacteria present in the human oral cavity, alongside the insufficient physical and mechanical strength of PMMA materials, an imperative arises to enhance the antibacterial properties of PMMA materials without compromising their physical and mechanical attribute.

In recent years, nano-antibacterial materials, notably nano-silver, have garnered considerable attention due to their broad-spectrum and enduring antibacterial effects without inducing bacterial resistance (Y. Liu et al., 2023;Y.-M. Wu et al., 2022). Research indicates that the incorporation of nanoparticles, which contain Ag, into PMMA materials can augment not only the antibacterial prowess of the PMMA materials but also enhance their mechanical properties, thus aptly fulfilling clinical requirements ( Bacali et al., 2020; Oyar et al., 2018; Garcia et al., 2021; Chen et al., 2017; Alhotan et al., 2023). Recent studies indicate that incorporating metal nanoparticles, such as silver nanoparticles, into inorganic carriers can enhance nanoparticle dispersion in polymers and mitigate the aggregation of these nanoparticles due to surface effects. This improvement facilitates the optimal utilization of the antibacterial properties of silver nanoparticles (Liu et al., 2019). Common carriers for nano-silver include zinc oxide (Pham et al., 2021), silica (Wong et al., 2017), montmorillonite (Fernández Solarte et al., 2019), zeolite (Youssef et al., 2019), graphene oxide (Huong et al., 2020), and zirconium phosphate (Liu et al., 2022). Researchers have investigated the incorporation of zinc oxide (Mousavi et al., 2020), silicon dioxide (Aati et al., 2022), graphene oxide (Arslan et al., 2023), and zeolite (Malic et al., 2019), all containing nano-silver, into PMMA materials. These studies explore the influence on the antimicrobial and mechanical properties of PMMA, offering new insights for clinical enhancements of these properties. However, zirconium α-phosphate, distinct from the carriers mentioned earlier, exhibits a two-dimensional layered nanosheet structure. It possesses exceptional ion exchange capabilities, catalytic activity, stable physicochemical properties, and biosafety, making it a versatile carrier for various medicines with significant potential in biomedicine (Saxena et al., 2013; Ramos-Garcés et al., 2021; Nocchetti et al., 2022). Moreover, the layered structure of zirconium phosphate nanosheets allows for increased surface area upon exfoliation, enabling a higher loading capacity for silver nanoparticles and thus enhancing the material’s antibacterial efficacy (Nocchetti et al., 2022). Despite these advances, research on the effects of nano-Ag-ZrP in PMMA materials, particularly regarding their antibacterial and mechanical properties, remains limited, suggesting an area ripe for further investigation.

Consequently, this study employs commercially available nano Ag-ZrP (RHA-1F-II) as the research subject, integrating it with room temperature curing PMMA material for material characterization analysis, and examines the impacts of nano Ag-ZrP antibacterial agent on the antibacterial attributes, mechanical properties, and biosafety of the room temperature curing PMMA material.

## 2 Materials and methods

### 2.1 Materials

Self-setting denture base polymer (NISSIN, Japan); self-setting denture water (NISSIN, Japan); Nano-Ag-ZrP (RHA-1F-II, China); BHI broth medium (Hopebio, China); LB broth medium (Hopebio, China); Nutritional Agar (Sigma, USA); SYTO 9/PI Live/Dead Bacterial Staining Kit (Thermo Fisher, USA); DMEM culture medium (Gibco, USA); Fetal Bovine Serum (FBS; Gibco, USA); 0.25% trypsin (Gibco, USA); Cell Counting Kit-8 (CCK-8); Calcein-AM/PI Live/Dead Cell Staining Kit (Beyotime, China); Annexin V-FITC/PI Apoptosis Detection Kit (Beyotime, China).

### 2.2 Preparation of specimen

PMMA powder and nano-Ag-ZrP were amalgamated in predefined mass fractions, subsequently subjected to an 8-hour processing in a planetary ball mill to procure the mixed powder.The material of the ball milling tank is zirconia, with a volume of 50 ml and a working volume of 15 ml;The material of the grinding ball is zirconia, with a total mass of 50 g, a diameter of 5 mm, and a ball to material ratio of 5:1; The working speed of the ball mill is 180 rpm/min. The requisite mass fractions of nano-Ag-ZrP for experimentation were defined as 0wt%, 0.5wt%, 1.0wt%, 1.5wt%, 2.0wt%, 2.5wt%, and 3.0wt%. Through combining the mixed powders with denture water, samples of disparate shapes and sizes were fabricated, culminating in the formation of nano-Ag-ZrP/PMMA materials. Conforming to relevant standards (ISO 22196:2007; ISO 20795-1:2008; ISO 527:2012), the antimicrobial specimen dimensions were set at (50 ± 2) mm × (50 ± 2) mm × (2 ± 0.5) mm; the three-point bending specimen at 64 mm × (10.0 ± 0.2) mm × (3.3 ± 0.2) mm; the modified three-point bending specimen at 39 mm × (8.0 ± 0.2) mm × (4.0 ± 0.2) mm, featuring a central notch measuring (3.0 ± 0.2) mm in length and 0.5 mm in width. The tensile test specimen adhered to a 5B dumbbell shape, with an overall length of 35.0 mm, end width of (6.0 ± 0.5) mm, narrow section width of (2.0 ± 0.1) mm, and a thickness of 1.0 mm.

### 2.3 Nano Ag - ZrP characterization

Surface morphology and elemental mapping of nano-Ag-ZrP were scrutinized utilizing field emission scanning electron microscopy (SEM;FEI, nova nanosem650,America) and transmission electron microscopy(TEM;FEI,F20,America). Additionally, the functional groups, crystal compositions, and valence states of Ag-ZrP were characterized through Fourier transform infrared spectroscopy(FT-IR;Nicolet,iS10,America),X-ray diffraction(XRD;Bruker,D8,Germany), and X-ray photoelectron spectroscopy(XPS; Thermo Fisher, ESCA LAB 350Xi,America).

### 2.4 The separation, culture and characterization of primary HGFs

Ethical approval was obtained from the Ethics Committee of the Stomatology Hospital of Jilin University. Gingival tissue, procured from patients aged 18-28 undergoing mandibular third molar extraction, necessitated the adjacent mandibular third molar to be healthy. Upon acquiring patient consent, samples were harvested around the extraction wound. Primary Human Gingival Fibroblasts (HGFs) were isolated and cultured utilizing the tissue block method. Optimal efforts were exerted to remove the epithelial tissue utilizing ophthalmic scissors. Gingival tissue, post-epithelial removal, was subjected to 50 repeat washes with DMEM medium, supplemented with 400 U/mL penicillin and 0.4 mg/mL streptomycin, until overt blood stains and impurities were absent. The tissue was then dissected into approximately 1 mm³ tissue blocks using ophthalmic scissors within a 60 mm culture dish. Subsequently, the tissue blocks were uniformly seeded into the base of T25 culture flasks with an approximate spacing of 0.5 cm between each block. Each flask was inoculated with about 10 tissue blocks, complemented by 5 ml of DMEM medium enriched with 20% Fetal Bovine Serum (FBS). The culture flasks were then incubated at 37°C, 5% CO_2_ for 4 hours, following which, the flasks were gently inverted and returned to the incubator. Minimal disturbance of the culture flasks was endeavored for the initial 3 days, with medium changes executed every 3 days. Once the primary cells occupied 60~70% of the flask bottom, the first passage was conducted. HGFs from passages 4~6 were designated for experimental use. Characterization of the isolated HGFs was accomplished through immunohistochemistry and immunofluorescence.

### 2.5 Detection of biosafety

#### 2.5.1 Preparation of specimen extract

The specimen was fashioned into a 10 mm x 10 mm x 2 mm round sheet. Adhering to ISO 10993-12:2012, extracts were formulated at an extraction ratio of 3.0 cm²/mL. For each group, samples were positioned within centrifuge tubes, accompanied by 733 μl of DMEM culture medium, which contained 10% FBS and 1% dual antibodies. Subsequently, the centrifuge tubes were incubated at 37°C for a duration of 72 hours. Extracts were thereafter collected and reserved for subsequent utilization.

#### 2.5.2 Detection of cytotoxicity by CCK-8

Three 96-well culture plates were seeded with 1.0×10^4^ cells per well and incubated at 37° for 24 hours. Subsequently, the culture medium was aspirated, and wells were rinsed with PBS before the addition of 100 μl of extract from each specimen group to the respective wells. On the conclusion of the 1st, 2nd, and 3rd days, a well plate was selected, the spent culture medium was discarded, and 100 μl of CCK-8 culture medium was introduced to each well. Following a 1-hour incubation period, absorbance (OD) values were measured using an enzyme-linked immunosorbent assay apparatus, and the relative cell proliferation rate (RGR) was calculated utilizing the following formula:


$$\text{RGR}=\left(\text{O}{\text{D}}_{\text{control}}/\text{O}{\text{D}}_{\text{samples}}\right)\times 100\%$$

where OD_samples_ represents the absorbance value of each extract group, and OD_control_ denotes the absorbance value of the negative control group.

#### 2.5.3 Live and dead cell staining test

Cells were seeded into 6-well plates at a density of 1.0x10^5^ cells per well and incubated for 24h. Subsequently, the culture medium was aspirated, and 2 ml of extract for each group was added for further culturing. After an additional incubation of 72h, the extract was removed from each well, followed by the addition of Calcein-AM/PI staining solution, and a subsequent 30-minute incubation at room temperature, protected from light, ensued. Observations were conducted using a fluorescence microscope.

#### 2.5.4 Annexin V-FITC/PI flow cytometry was used to detect apoptosis

Cells were cultured in 6-well plates at a density of 1.0x10^5^ cells per well and incubated for 24h. Following incubation, the culture medium was aspirated and wells were washed with PBS; subsequently, extracts for each group were introduced and maintained for an additional 72h. Cells were then harvested by centrifugation, and Binding Buffer was utilized to resuspend the cells, adjusting the cell concentration to 1.0x10^6^/mL. A 100 μl aliquot of cell suspension was transferred to a centrifuge tube, and Annexin V-FITC and PI dyes were added according to the manufacturer’s instructions. The mixture was incubated for 20 min in dark conditions, followed by analysis via flow cytometry.

### 2.6 Detection of antibacterial property

#### 2.6.1 Preparation of bacterial suspensions

*S. mutans* (ATCC 700610) and *E. coli* (ATCC 25922) were revitalized. Individual colonies of *S. mutans* and *E. coli* were isolated and cultured in Brain Heart Infusion (BHI) and Lysogeny Broth (LB) media, respectively, and incubated at 37°. Following an 8-hour incubation period, the optical density (OD) of the bacterial solutions was ascertained using a spectrophotometer. Subsequently, the concentrations of the bacterial suspensions were standardized to 1.0×10^6^ CFU/ml to prepare working bacterial suspensions.

#### 2.6.2 The antimicrobial rate of each group was determined by film coating method

Both sides of the specimens were exposed to ultraviolet light for 12 hours, followed by transfer to sterile petri dishes on an ultra-clean workbench. Working bacterial droplets of *S. mutans* and *E. coli*, each measuring 100 μl, were absorbed and introduced to the surface of each specimen group. Polyethylene film was applied to the surface of each specimen group, ensuring close contact with the specimen, and then incubated at 37° for 24 hours. Subsequently, the specimens and films were cleaned with 20 ml of 0.85% NaCl solution, and the collected eluent was vigorously shaken and diluted in a two-fold ratio. The diluted eluent, 100 μl, was plated and incubated at 37°. After 36 hours, colonies were enumerated on the agar plates. The antimicrobial rate was calculated using the formula R=(B-C)/B, where R represents the antibacterial rate, B is the average colony count of specimens in the 0%wt group (CFU/ml), and C denotes the average colony count in the experimental group (CFU/ml).

#### 2.6.3 SYTO 9/PI staining of live and dead bacteria

The size of the specimens adhered to the dimensions specified in Section 2.5.1. Specimens from each group were positioned in 48-well plates, with each well receiving 500 μL of a working bacterial suspension of *S. mutans* and *E. coli* for a 24-hour incubation period. Subsequently, the bacterial solution from each well was aspirated and the specimens were rinsed with 0.85% NaCl solution. Specimens from each group were then transferred to centrifuge tubes, combined with 3 ml of 0.85% NaCl solution, and the bacteria from the specimen surfaces were collected following an ultrasonic shock for 5 minutes. Bacterial solutions for each group were centrifuged at 10000 g for 10 minutes; thereafter, the supernatant was discarded, and 200 μL of staining solution was added to suspend the bacterial pellet, followed by incubation in the dark at room temperature for 15 minutes. Finally, 5 μL of the bacterial solution was extracted from each tube, applied to a slide, and observed via a fluorescent inverted microscope.

#### 2.6.4 Bacterial adhesion test

The specimen size and the co-culture methodology with the bacterial solution adhered to the parameters set in Section 2.6.3. Following a 24-hour period, the 48-well plate was removed, rinsed with 0.85% NaCl solution, and subsequently exposed to 2.5% glutaraldehyde, being fixed at 4° for 1 hour in light-protected conditions. Each well was aspirated of the glutaraldehyde solution and sequentially dehydrated with ethanol solutions of increasing concentrations (30%, 50%, 70%, 90%, and 100%) for 10 minutes each. Subsequently, the surface of the specimens in each group underwent gold sputter coating, and bacterial adhesion on the surfaces was observed using Scanning Electron Microscopy (SEM).

### 2.7 Mechanical property test

#### 2.7.1 Three-point bending test

In adherence to the YY 0270.1-2011 standard, the test span was defined as (50 ± 0.1 mm) and the indenter speed was established at 5.0 mm/min. The bending strength, *σ*, is calculated as follows:


$$\sigma =3FL/2bh^{2}$$

where: *σ* represents the bending strength in MPa; *F* signifies the maximum force exerted on the specimen, with a unit of N; *L* indicates the span and is measured in mm; *b* denotes the width of the specimen in mm; *h* defines the height of the specimen in mm. Similarly, the bending elastic modulus, *E*, can be calculated using the formula:


$$E=3KL^{3}/4bh$$

where: *E* represents the bending elastic modulus in MPa; *K* signifies the maximum slope in the stress-strain curve, expressed in N/mm; *L*, *b*, and *h* retain their previously defined meanings.

#### 2.7.2 Improved three-point bending test

The span was established at (32.0 ± 0.1) mm, and the indenter speed was set to 1.0 mm/min. The formulas used to calculate the maximum stress intensity factor, *K_max_*, and the total breaking work, *W_t_*, are as follows:


$$K_{\max}=\frac{fP\max lt}{\left(btht^{3/2}\right)}\times \sqrt{10^{-3}}\;\text{MPa}\;{\text{m}}^{1/2}$$

$$\text{ f}\left(x\right)=3x^{1/2}\left[1.99-x\left(1-x\right)\left(2.15-3.93x+2.7x^{2}\right)\right]/\left[2\left(1+2x\right){\left(1-x\right)}^{3/2}\right]$$

x=a/h

*P_max_* represents the maximum load exerted on the specimen and is measured in N; a delineates the total length of the specimen notch, with units in mm; *h_t_* is the specimen’s height, in mm. Additionally, *b_t_* signifies the specimen’s width and is also measured in mm, while lt designates the test span, expressed in mm.


$${\text{ W}}_{\text{t}}=\frac{U}{\left[2b_{t}\left(h_{t}-a\right)\right]}\times 1000\;\text{J}/{\text{m}}^{2}$$

U represents the area documented under the load/deflection curve, measured in N·mm. The total length of the specimen notch is denoted by *a* and is expressed in mm. The height and width of the specimen are represented by *h_t_* and *b_t_* respectively, both quantified in mm.

#### 2.7.3 Tensile strength test

Conforming to ISO 527:2012, a 5B sample was utilized, establishing a fixture distance of (20 ± 2)mm and a standard distance of (10 ± 0.2)mm, with a test speed set at 1 mm/min. The tensile strength, *β*, is defined as *β*=F/A, where *β* is the tensile strength in MPa; F signifies the critical failure force value of the specimen, measured in N; and A represents the cross-sectional area of the sample, expressed in mm^2^.

### 2.8 Statistical analysis

Data from each group underwent statistical analysis utilizing GraphPad Prism. Here,`X denotes the mean of the data, while S signifies the standard deviation. Subsequent to the normal distribution test and variance homogeneity test, a one-way analysis of variance, coupled with the LSD-t test, was employed for analyzing statistical differences. The P-value threshold was established at 0.05, with P< 0.05 indicating statistical significance in the observed differences.

## 3 Results and discussion

### 3.1 Characterization of nano-borne Ag-ZrP

**Figure 1:**
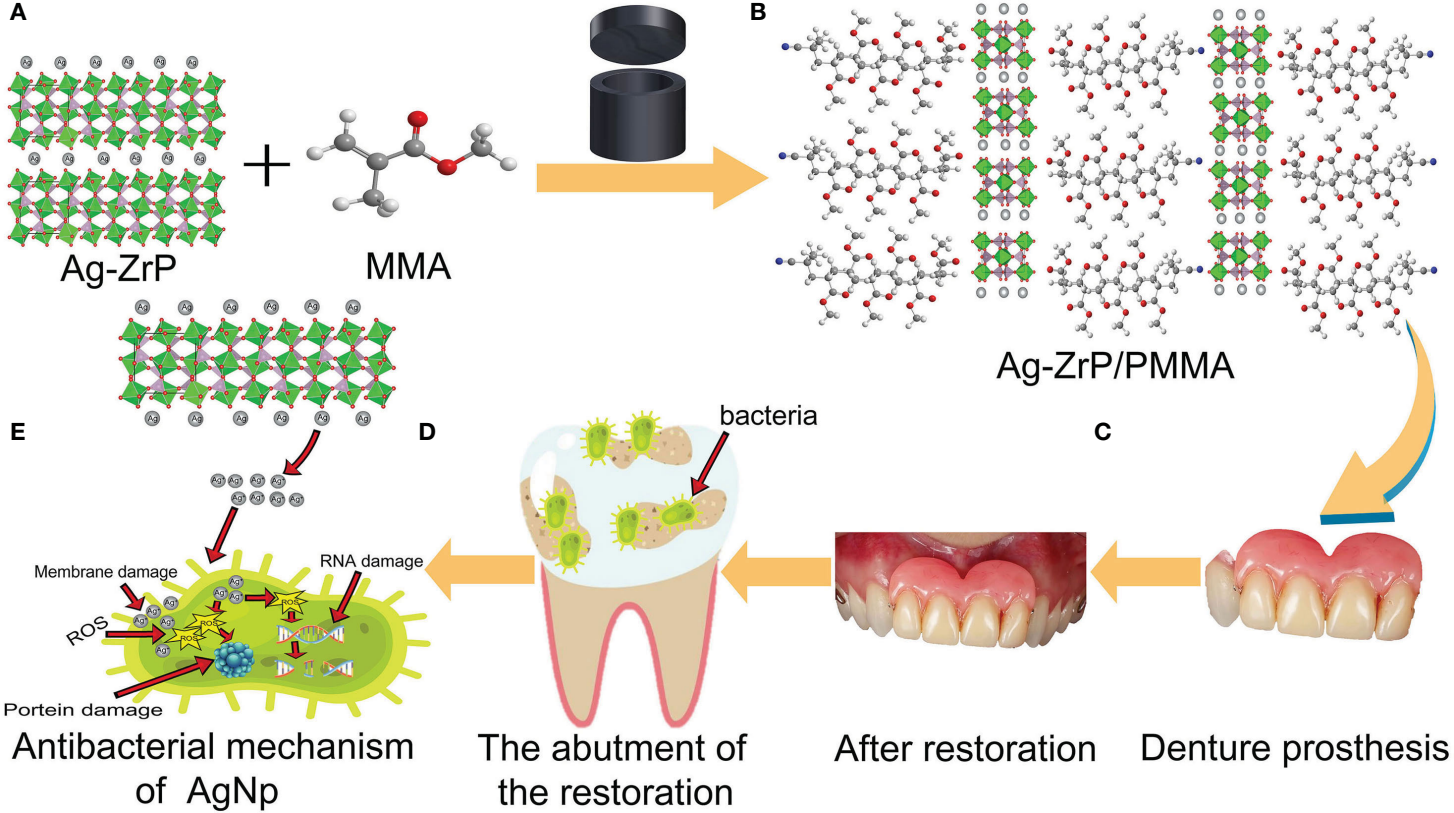
**(A, B)** is the schematic diagram of adding nano-Ag-ZrP to PMMA material; **(C)** is a denture prosthesis made of nano-Ag-ZrP/PMMA material; **(D)** for restoration abutments that are not easy to clean and adhere to bacteria; **(E)** is release Ag^+^ for nano-Ag-ZrP and produces antibacterial effect.

**Figure 2:**
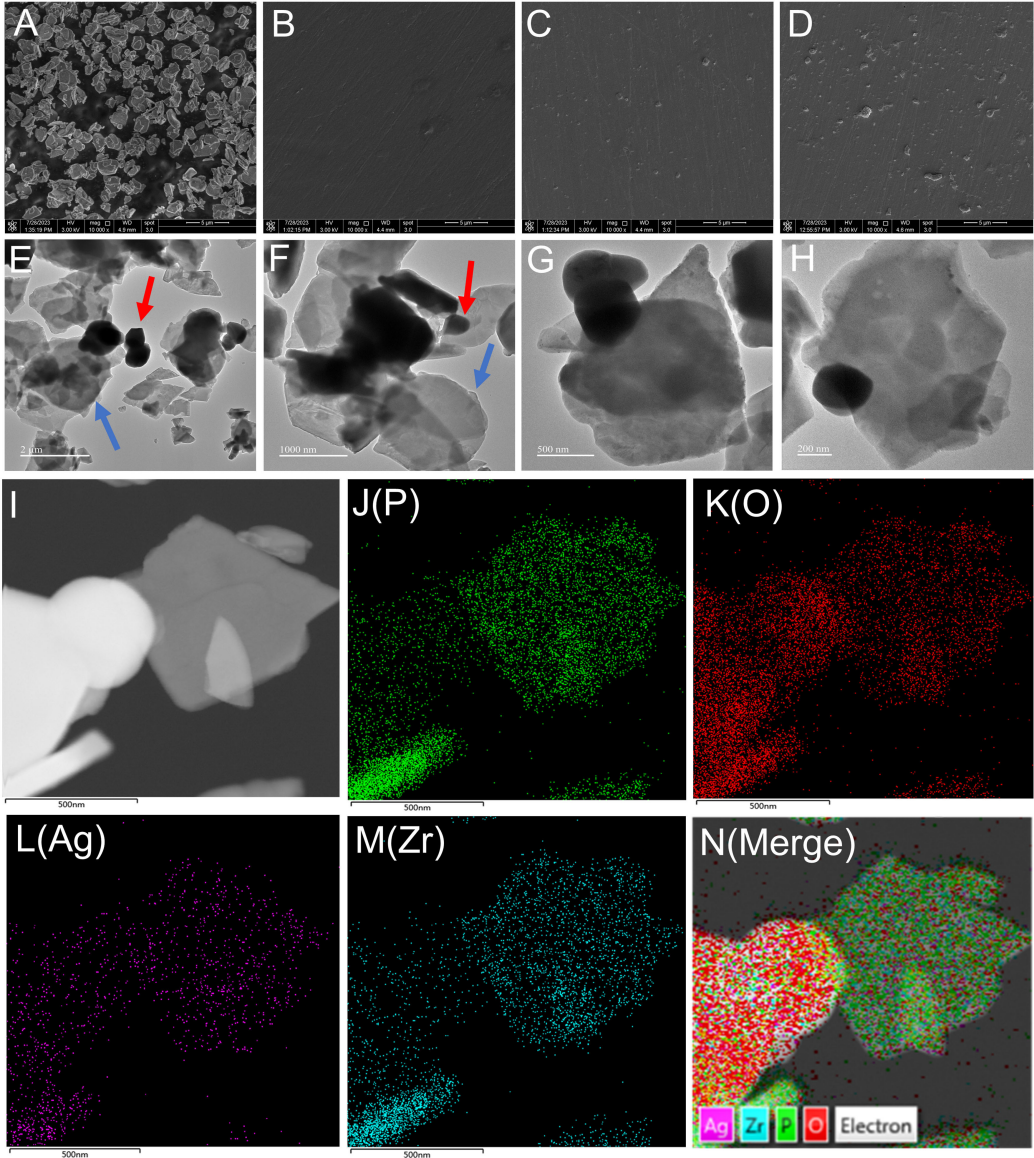
**(A)** is the SEM image of nano-Ag-ZrP; **(B-D)** are the SEM images of nano-Ag-ZrP/PMMA materials with 0%wt, 1.5%wt and 3.0%wt added in sequence. **(E-H)** is TEM image of nano-Ag-ZrP; **(I-N)** is the TEM-EDS element mapping of P, O, Ag and Zr of nano-Ag-ZrP.

**Figure 1** shows the synthesis of nano-Ag-ZrP/PMMA composites and the antibacterial mechanism of AgNp. SEM (Loaded voltage is 3kV,and sputtered with Au) reveals that nano-Ag-ZrP exhibits a layered cubic structure (**Figure 2A**), while **Figures 2B-D** sequentially depict PMMA materials with the incorporation of 0%wt, 1.5%wt, and 3.0%wt Ag-ZrP, respectively. Notably, in the 1.5%wt group, nano-Ag-ZrP minimally agglomerates within PMMA, showcasing a relatively uniform distribution, whereas the 3.0%wt group experiences an elevation in Ag-ZrP aggregation. It is speculated that the agglomeration of nano-Ag-ZrP in the 3.0%wt group was increased because the excessive addition of nano-Ag-ZrP led to the increase of the distribution density of nano-Ag-ZrP in PMMA materials, and the enhancement of the electrostatic attraction and van der Waals force, which led to the increase of the agglomeration of nano-Ag-ZrP particles (Sun et al., 2019; Kignelman et al., 2021; Kumar et al., 2022). **Figures 2E-H** present images of Ag-ZrP, as observed through TEM (Loaded voltage is 200KV,and loaded on copper mesh), at magnifications of 2 μm, 1 μm, 500 nm, and 200 nm, respectively. The ZrP crystal, featuring a sheet structure, is indicated by a blue arrow, and nano silver, exhibiting a black spherical shape, is indicated by a red arrow. Observable from the figure is the loading of nano-Ag particles onto the ZrP surface, bearing substantial morphological resemblance to the nano-Ag-ZrP detailed in existing literature (Qin et al., 2023; Yoshihara et al., 2021; Nocchetti et al., 2022). According to the TEM-Energy Dispersive Spectroscopy (TEM-EDS) element mapping analysis (**Figures 2I-N**) for P, O, Ag, and Zr elements, an intuitive reflection of each element’s distribution within nano-Ag-ZrP is provided, illustrating a uniform dispersion of nano-silver.

**Figure 3:**
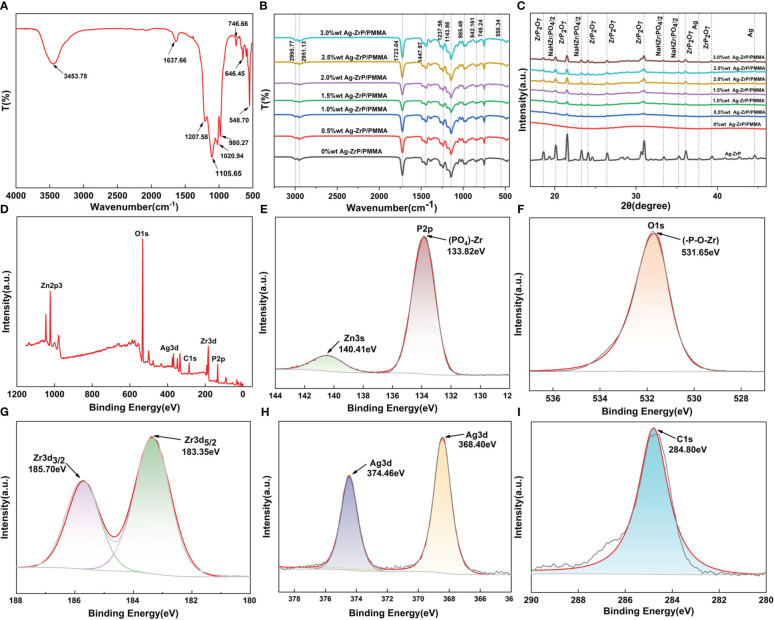
**(A)** is the FT-IR diagram of nano-Ag-ZrP; **(B)** is the FT-IR diagram of nano-Ag-ZrP/PMMA composites across different groups. **(C)** is the XRD analysis diagram of nano-Ag-ZrP and different groups of nano-Ag-ZrP/PMMA composites; **(D)** is the total XPS spectrum of nano-Ag-ZrP; **(E-I)** are the XPS spectra of P, O, Zr, Ag and C elements.

**Figure 3A** displays the FT-IR (400 cm^-1^~ 4000 cm^-1^) spectroscopy analysis of Ag-ZrP, where the peak identified at 3453.78 cm^-1^ corresponds to the symmetric stretching vibration of OH, and the peak at 1637.66 cm^-1^ is attributed to the stretching vibration of OH from water molecules situated between crystal layers ( Bhatt et al., 2019). Peaks detected at 1207.58 cm^-1^ and 980.27 cm^-1^ associate with in-plane and out-of-plane vibrations of P-OH groups, respectively ( Safari et al., 2016), while those at 1020.94 cm^-1^ and 1105.65 cm^-1^ represent symmetric stretching vibrations of P-O bonds (Yan-Qing et al., 2015). The peak at 746.66 cm^-1^ pertains to P-O-P stretching vibrations(Sinhamahapatra et al., 2010) whereas those at 646.45 cm^-1^ and 548.70 cm^-1^ denote tensile vibrations of Zr-O bonds (Yan-Qing et al., 2015; Xu et al., 2020). Nano-Ag-ZrP was incorporated into PMMA materials in varying mass fractions. The FT-IR spectra for each nano-Ag-ZrP/PMMA composite are presented in **Figure 3B**. The spectra exhibit peaks at 2995.77 cm¹ and 2951.13 cm¹, corresponding to the asymmetric vibration of CH_3_ groups (Abutalib and Rajeh, 2020). Additional characteristic peaks include 985.49 cm¹ for C-C bond stretching, 1143.98 cm¹ for CH_3_ bending vibration, 1237.56 cm¹ for C-O bond stretching, 1447.97 cm¹ for CH_3_ deformation, and 1723.04 cm¹ for C=O bond stretching (Duan et al., 2008). The peak at 842.16 cm¹ is identified as the C=O bond (Abutalib and Rajeh, 2020), with in-plane and out-of-plane flexural vibrations of the C=O bond at 749.24 cm¹ (Singhal et al., 2013). Notably, a peak at 550.34 cm¹, attributed to the Zr-O bond stretching, appears in the spectra (Xu et al., 2020). For compositions with nano-Ag-ZrP, a new peak at 550.34 cm¹ emerges, becoming increasingly prominent with higher nano-Ag-ZrP concentrations, reaching its maximum at 3.0%wt. These results confirm the successful integration of nano-Ag-ZrP into PMMA materials, with a gradual increase in nano-Ag-ZrP content from 0%wt to 3.0%wt.XRD analysis (The XRD test uses copper targets with a scanning range of 5~70 degrees and a speed of 10 degrees per minute) of nano-Ag-ZrP and each group of nano-Ag-ZrP/PMMA composites (**Figure 3C**) indicates that peaks at 2θ values of 18.59°, 21.53°, 24.09°, 26.43°, 30.95°, 36.04°, and 39.28° correspond to ZrP_2_O_7_ (PDF No. 00-010-0004). Characteristic peaks at 20.12°, 23.27°, 33.38°, and 35.24° are aligned with NaHZr(PO_4_)_2_ (PDF No. 00-055-0206), while those at 37.72° and 44.57° correspond to characteristic peaks of nano silver (PDF No. 04-003-1659). The XRD results for the nano-Ag-ZrP/PMMA composites across different groups reveal a correlation between the concentration of nano-Ag-ZrP and the prominence of characteristic diffraction peaks. Specifically, as the nano-Ag-ZrP content increases, the distinct diffraction peaks corresponding to ZrP_2_O_7_, NaHZr(PO_4_)2, and Ag become increasingly pronounced. These peaks are most pronounced in the 3.0%wt group, substantiating the successful integration of nano-Ag-ZrP into the PMMA materials.XPS (The results of XPS were analyzed by Avantage software) global spectrum analysis of nano-Ag-ZrP is presented in **Figure 3D**. The binding energies at 1022.80 eV and 1044.52 eV correspond to Zn2P_1/2_ and Zn2P3 orbits, respectively (Mahakal et al., 2023). According to the reagent manufacturer, the Ag-ZrP antibacterial agent incorporates a minimal quantity of zinc oxide (approximately 5.0~10.0%wt), primarily functioning as a dispersant, and the peak at 248.80 eV signifies the charge correction peak of C1s. **Figures 3E-I** sequentially exhibit the XPS spectra of P, O, Zr, Ag and C elements. The figure highlights that the peak of P2p at 133.82 eV corresponds to P^5+^, representing the (PO_3_)-Zr bond (Yan-Qing et al., 2015; Yuan et al., 2005). while the peak at 140.41 eV emerges as the interference peak of Zn3s. The peak of 531.65 eV aligns with O1s, corroborating the binding energy of the (-P-O-Zr) bond (Pourbeyram, 2016). **Figure 3G** elucidates the XPS map of Zr, where Zr3d demonstrates two distinctive peaks: 183.35 eV representing Zr3d_5/2_ and 185.70 eV indicative of Zr3d_3/2_, denoting Zr^4+^ and indicating covalent connections between Zr^4+^ and PO4^3-^ as substantiated by related research (Yan-Qing et al., 2015; Chen et al., 2022; Li et al., 2021). Additionally, AgNP exhibits two prominent peaks, with 368.40 eV pertaining to Ag3d_5/2_ and 374.46 eV to Ag3d_3/2_(Xu et al., 2020; Biswal et al., 2011). The comprehensive analysis through SEM, TEM, FT-IR, XRD, and XPS preliminarily substantiates that the material can be classified as nano-Ag-ZrP,and Ag-ZrP has been successfully integration of nano-Ag-ZrP into the PMMA materials.

### 3.2 Characterization and identification of primary HGFs

**Figure 4:**
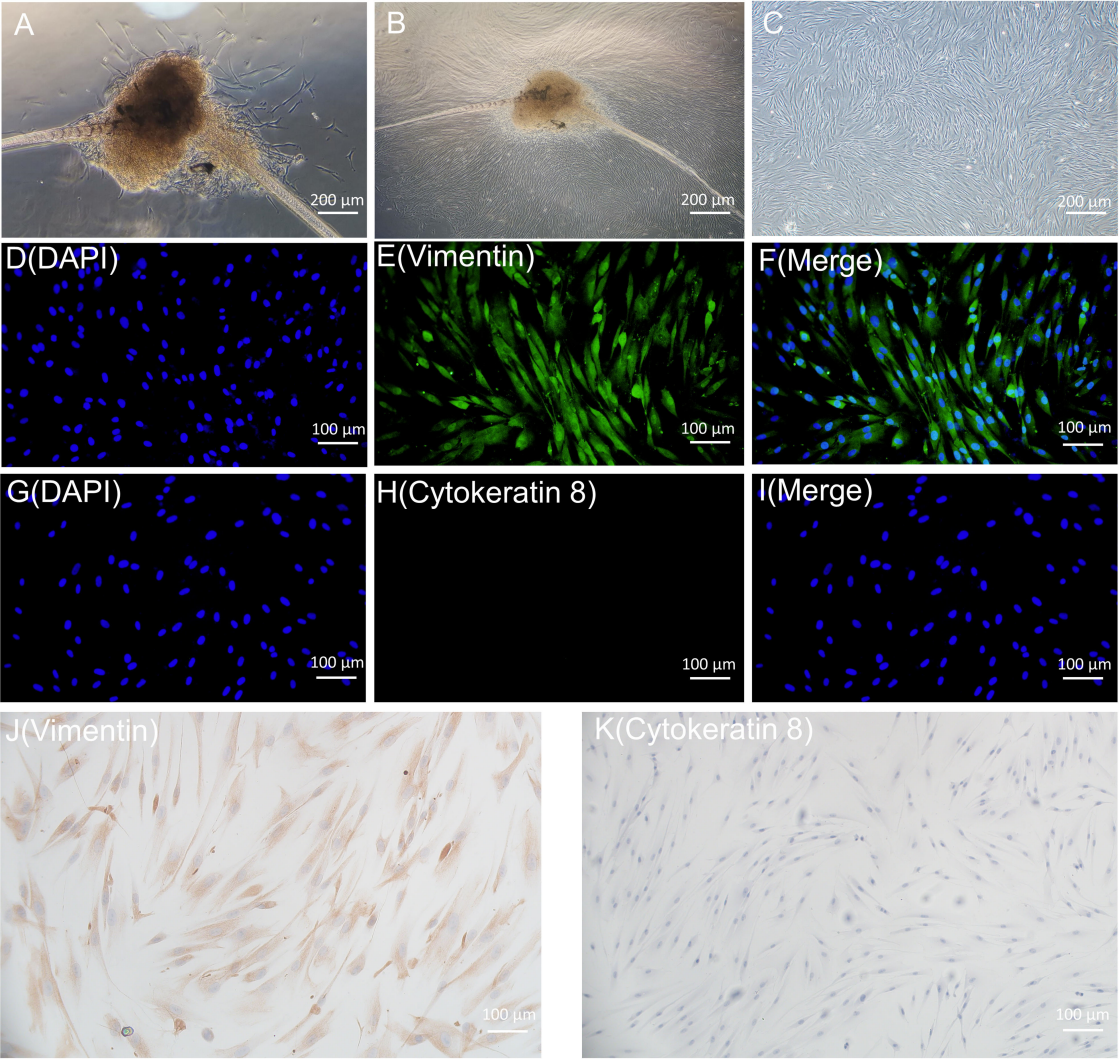
**(A, B)** are the primary HGFs flowing out of tissue blocks. C is the microscopic morphology of 5th generation HGFs. **(D-I)** is the result of immunofluorescence staining of HGFs; **(J, K)** were immunohistochemical staining results of HGFs.

On day 8, primary HGFs were observed to migrate from the gingival tissue (**Figure 4A**), and by day 15, the cells exhibited a swirl-like arrangement (**Figure 4B**). **Figure 4C** illustrates the morphology of fifth-generation HGFs under the microscope (40x magnification), revealing spindle- or star-shaped cells with centrally located nuclei, clear nucleoli, and round or oval nuclear shapes, consistent with typical fibroblast characteristics. Immunostaining was performed on the fifth-generation HGFs utilizing anti-vimentin antibody and anti-cytokeratin antibody (Cytokeratin 8). **Figures 4D-I** display immunofluorescence staining, while **Figures 4J, K** depict immunohistochemical staining. Staining for Vimentin was positive, whereas Cytokeratin 8 staining was negative. These results align with the staining findings for HGFs by Xuqian Liu, Kai Yang et al., confirming the cells to be mesodermal-origin fibroblasts (Liu et al., 2016; Yang et al., 2021).

### 3.3 Effect of nano-Ag-ZrP on biosafety of room-temperature cured PMMA materials

**Figure 5:**
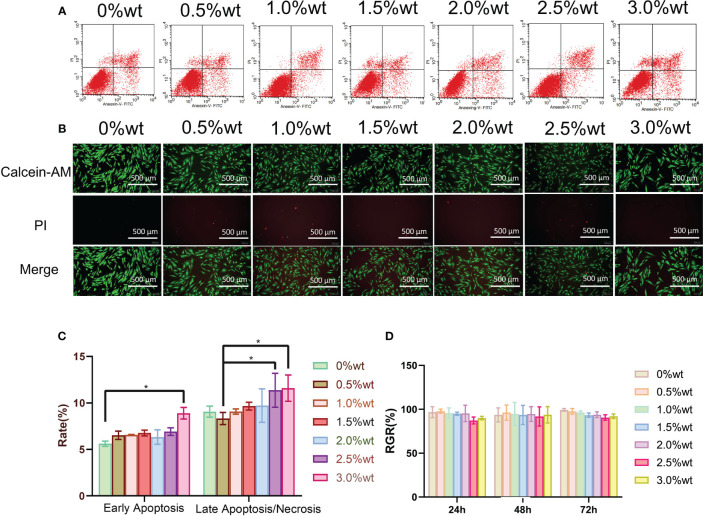
**(A)** is the Annexin V-FITC/PI double staining was used to detect apoptosis. **(B)** is the staining results of live and dead cells after incubation with HGFs 3 days in the extract solution of each group. **(C)** is the analysis of apoptosis detection results; **(D)** is the detection result of CCK-8 method. * symbol 0.01<P-value<0.05,it is indicating significant differences.

AgNP has been documented to exert specific impacts on cell activity, oxidative stress, and cytoskeletal protein among other factors (Shimizu et al., 2022; Perde-Schrepler et al., 2019) necessitating further investigation into the biosafety of Ag-ZrP/PMMA materials. **Figure 5A** illustrates the outcomes of apoptosis detection via Annexin V-FITC/PI double staining, following a 3-day culture of HGFs with the extract from each sample group. Subsequent statistical analysis of the apoptosis findings (**Figure 5C**) revealed a notable increase in the early apoptosis rate of HGF-s in the 3.0%wt group compared to the 0%wt group (P< 0.05). In comparison to the 0.5%wt group, the late apoptosis/necrosis rates of HGFs in the 2.5%wt and 3.0%wt groups notably escalated (P< 0.05), yet remained statistically indistinguishable among other groups. A plausible reason for the augmented apoptosis rate in 2.5%wt and 3.0%wt groups could be attributed to a minor release of AgNp with the elevation of Ag-ZrP supplemental level, thereby inducing a modest cytotoxicity. Despite this, the majority of cells maintained normal activity, and the overarching apoptosis rate did not reach substantial levels, warranting the incorporation of additional detection methods for accurate determination. **Figure 5B** portrays the staining results of live and dead cells post a 3-day culture of HGFs in each group’s extract solution, demonstrating that an uptick in nano-Ag-ZrP supplemental level correlated with an elevation in the count of live cells across all experimental groups and a reduction in the proportion of dead cells, without overt cell morphological alterations across groups. The CCK-8 method, employed to ascertain the activity of HGFs post a 3-day culture in extracts from each group (**Figure 5D**), yielded no significant discre-pancy in the Relative Growth Rate (RGR) of HGFs among extract groups (P > 0.05), with an RGR of HGFs exceeding 85% following 24h, 48h, and 72h of culture. According to the cytoto-xicity grading evaluation criteria stipulated by ISO 10993-5:2009, the cytotoxicity of extracts from each group post 24h, 48h, and 72h of cultured HGFs consecutively could be classified as less than or equal to grade 1, with no discernible cytotoxicity detected. Erkose and Jie SUN et al. incorporated AgNP into PMMA denture base materials, while Cecilia et al. introduced AgNP-GO into PMMA denture base materials, with neither finding substantial cytotoxicity in PMMA materials containing a low mass fraction of AgNP (Bacali et al., 2020; Kurt et al., 2017; Sun et al., 2021). Consequently, it may be inferred that the biosafety of roomtemperatur-e cured PMMA materials remains favorable when the addition of nano-Ag-ZrP is restrained below 3.0%wt.

### 3.4 Effect of nano-Ag-ZrP on antibacterial properties of room-temperature cured PMMA

**Figure 6:**
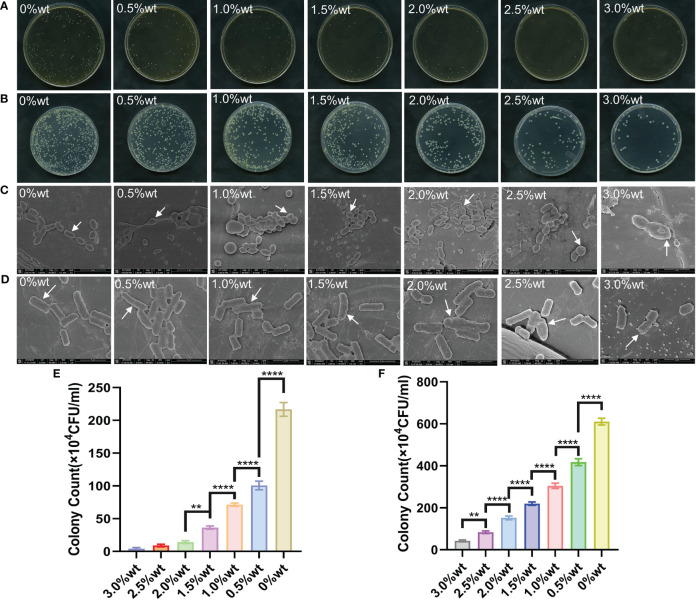
**(A)** is the plate colony count result of *S. mutans*. **(B)** is the colony count result of *E. coli*. **(C)** is the SEM image of *S. mutans* on the surface of each group of specimens. **(D)** is the SEM image of *E. coli* on the surface of each group of specimens; **(E)** is the results of statistical analysis of the colony count of *S. mutans*. **(F)** is the result of statistical analysis of *E. coli* colony count. * symbol 0.01<P-value<0.05, it is indicating significant differences; ** symbol 0.001<P-value<0.01, it is indicating that the difference is very significant; **** symbol P-value<0.0001, it is indicating that the significance of the difference is the highest.

**Table 1** illustrates the colony recovery number and antibacterial rate of *S. mutans* and *E. coli* across varied sample groups. The colony-counting results for *S. mutans* (**Figure 6A**) and *E. coli* (**Figure 6B**) elucidate that an elevation in nano-Ag-ZrP content correspondingly reduces the colony number of sample elutes across all groups. A diminution to the least colonies of *S. mutans* and *E. coli* is observed at 3.0%wt. **Figure 6E** reveals the statistical analysis results of *S. mutans* recovered colony numbers across different groups. A substantial reduction in the *S. mutans* colony number is observed in all groups compared to the control group (P< 0.05). Pairwise comparison among experimental groups reveals statistically significant discrepancies (P< 0.05) among them, save for the comparisons between 2.0%wt and 2.5%wt, 2.0%wt and 3.0%wt, and 2.5%wt and 3.0%wt (P > 0.05). For *E. coli*, statistical results of recovered colony numbers (**Figure 6F**) demonstrate a notable reduction in colony numbers across all experimental groups compared to the 0%wt control group (P< 0.05), with all experimental group pairwise comparisons showcasing statistically significant disparities (P< 0.05). Research by Shenggui Chen et al. involved the addition of 0.1-0.25%wt AgNP-CNCs (nanocellulose) to PMMA materials (Chen et al., 2018), Souza Neto et al. introduced 0.05%wt AgNP to PMMA resin material (Souza Neto et al., 2019), and Sultan Aati et al. incorporated silver-carrying mesoporous SO_2_ nanoparticles into the denture base (Aati et al., 2022). All studies affirm that materials embedded with AgNP can substantially enhance the antibacterial capability of PMMA materials, aligning with the findings obtained in this study that an increased AgNP content improves antibacterial effects. SEM images depicting *S. mutans* adherence to the surface of specimens in each group are presented in **Figure 6C**. *S. mutans* in the 0%wt∼0.5%wt group exhibits a smooth surface and a continuous, intact envelope(indicated by white arrows), contrasted by the 1.0%wt group where the envelope appears wrinkled and sunken. Within the 1.5% ~ 3.0%wt groups, the membrane of *S. mutans* evidences varied degrees of perforation and damage (indicated by white arrows). **Figure 6D** discloses SEM observation images of *E. coli* on specimen surfaces across groups. As you can see from the arrows,*E. coli* in the 0%wt∼0.5%wt group manifests well-preserved morphology, with a predominantly smooth surface and negligible folding and collapse. However, in the 1.0%wt and 1.5%wt groups, the *E. coli* cell membrane surface becomes notably rough and wrinkled. A further increase in the nano-Ag-ZrP antibacterial agent leads to an escalation in *E. coli* surface folds, cell membrane rupture, and exposure of bacterial contents in the 2.0% to 3.0%wt group. The SEM images of *S. mutans* and *E. coli* on the surface adhesion of specimens of each group showed that the damage degree of *S. mutans* and *E. coli* on the surface adhesion of specimens of each group was gradually serious with the increase of nano-Ag-ZrP addition. It is shown that the antibacterial properties of PMMA materials can be improved by adding nano-Ag-ZrP to PMMA materials. The presumed antibacterial mechanism may be attributed to direct contact between AgNP and the bacterial cell membrane, Ag^+^ release, bacterial cell wall and cell membrane disruption, reactive oxygen species (ROS) generation, bacterial substance leakage, and subsequent bacterial internal structure obliteration, culminating in a bactericidal effect (Ji et al., 2020; Hajipour et al., 2012; Dakal et al., 2016; Estevez et al., 2021).

**Table 1:** The number of *S. mutans* and *E. coli* colonies recovered and the antibacterial rate of each group.

Additive amount (wt, %)	Colony count (×10^4^CFU/ml,`X ± S)		Antibacterial rate (R,%)	
	*S. mutans*	*E. coli*	*S. mutans*	*E. coli*
0	216.70 ± 10.50	610.30 ± 16.26	–	–
0.5	100.70 ± 6.50	417.30 ± 16.50	53.53	31.62
1.0	71.33 ± 2.51	304.30 ± 12.58	67.08	50.14
1.5	36.33 ± 2.51	219.70 ± 8.15	83.23	64.00
2.0	14.33 ± 2.08	152.00 ± 9.00	93.38	75.09
2.5	9.00 ± 2.00	83.67 ± 6.11	95.85	86.30
3.0	4.33 ± 1.52	42.67 ± 4.04	98.00	92.98

**Figure 7:**
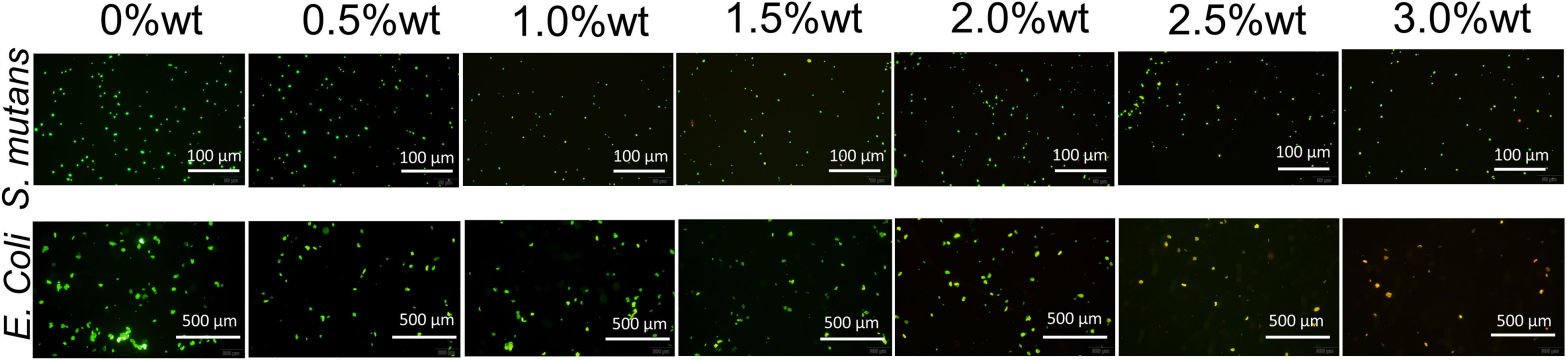
The staining results of *S. mutans* and *E. coli* on the surface of each group of specimens.

SYTO 9 has the capability to stain the nucleic acid of live bacteria, or bacteria possessing damaged cell membranes, resulting in green fluorescence emission, while propidium iodide (PI) can stain the nucleic acid of dead bacteria, or bacteria with compromised cell membranes, to emit red fluorescence. Upon concurrent application, bacteria with impaired cell membranes exhibit yellow fluorescence. **Figure 7** illustrates the staining results for live and dead bacteria of *S. mutans* and *E. coli*. It is evident from the figure that, concomitant with the augmentation of nano-Ag-ZrP antibacterial agent content, the quantity of live *S. mutans* and *E. coli* bacteria on the specimen surface progressively diminished, whereas the number of dead bacteria or bacteria with damaged cell membranes progressively escalated. When the addition of Ag-ZrP antibacterial agent reached 2.5%wt, and subsequently 3.0%wt, the dead bacteria or bacteria with compromised cell membranes pervaded almost the entire visual field on the specimen surface, aligning with the outcomes from the film coating experiment.

### 3.5 Effect of nano-Ag-ZrP on mechanical properties of PMMA materials

**Figure 8:**
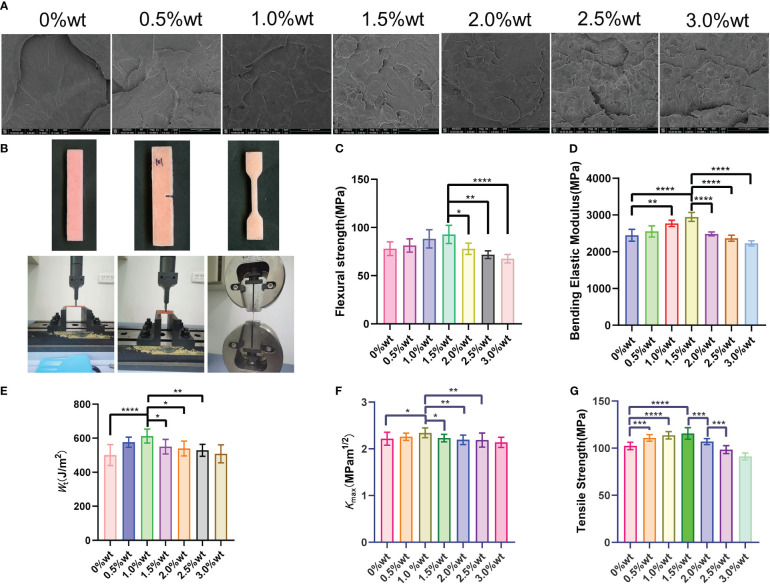
**(A)** is SEM observation of three-point bending specimen; **(B)** is the specimen shape and universal mechanical testing machine; **(C, D)** are the results of three-point curve test; **(E, F)** are the results of improved three-point bending test; **(G)** is the result of tensile test. * symbol 0.01<P-value<0.05, it is indicating significant differences; ** symbol 0.001<P-value<0.01, it is indicating that the difference is very significant; *** symbol 0.0001<P-value<0.001, it is indicating difference is more significant; **** symbol P-value<0.0001, it is indicating that the significance of the difference is the highest.

The fracture morphology of the sample, post-three-point bending test fracture, as visualized in the SEM image (**Figure 8A**), reveals that the cross-section of the sample in the 0%wt group is predominantly smooth with a flat, continuous fracture line, indicative of typical brittle fracture characteristics. With the escalation of nano-Ag-ZrP content, a gradual increase in the number of nano-Ag-ZrP crystals on the fracture surface of the 0.5%wt ~ 3.0%wt group is observable, and the evident rough stratification of the fracture surface displays dimple formation. Nano-Ag-ZrP particles were identified within the dimple, exhibiting classic ductile fracture traits. A notably deep and large dimple is present on the fracture surface of the 0.5%wt ~ 2.00%wt group. Conversely, the dimple on the fracture surface of the 2.5wt ~ 3.0%wt group is shallow and small, with Ag-ZrP crystal particles appearing more agglomerated. **Figure 8B** illustrates the shape of the mechanical properties test specimen and the universal mechanical testing machine. Three-point bending test results (**Figures 8C, D**) 1.5%wt, the bending strength and bending elastic modulus of the PMMA material incrementally rise, subsequently diminishing with a continuous increase of Ag-ZrP content. Both the bending strength and bending elastic modulus of the 1.5%wt group reached peak values. When compared with the 1.5%wt group, the 2.0%wt, 2.5%wt, and 3.0%wt groups exhibit significantly reduced bending strength and bending elastic modulus, with the difference being statistically significant (P< 0.05). **Figures 8E, F** depict the outcomes from the modified three-point bending test, illustrating a notable trend in the *W_t_* and *K_max_* of each group with varying nano-Ag-ZrP content. Specifically, in the range of 0%wt to 1.0%wt nano-Ag-ZrP, both *W_t_* and *K_max_* exhibit a gradual increment, showcasing a statistically significant difference in comparison to the 0%wt control group (P< 0.05). Conversely, in the 1.0%wt to 3.0%wt nano-Ag-ZrP range, a gradual decline in the *W_t_* and *K_max_* of the sample is observed, with the zenith being at the 1.0%wt group. The tensile test results from each group’s specimens (**Figure 8G**) demonstrate a gradual ascending trend in the tensile strength (TS) within the 0%wt to 1.5%wt group, peaking at 1.5%wt. Subsequently, a gradual descending trend in TS is exhibited by the experimental groups, nadiring at 3.0%wt. Rongrong CHEN et al. integrated 3.0wt% nano-Ag-ZrP (Novaron) into a blend comprising PMMA powder, MMA monomer, silanized nano-zirconia, and silanized aluminum borate whisker, determining that an enhancement in the bending strength and hardness of PMMA material was realized exclusively post nano-Ag-ZrP addition(R. Chen et al., 2017). Wenbo Liao et al., found that the addition of silanized nano-Ag-ZrP to PMMA material resulted in an initial increase followed by a decrease in the bending strength as the mass fraction of Ag-ZrP augmented from 0wt% to 3.0wt%. A pinnacle in the bending strength of PMMA material was achieved when the nano-silver zirconium phosphate was introduced at 1.0%wt (Liao et al., 2020). Incorporation of nanoparticles, such as zirconium dioxide (ZrO_2_), graphene oxide (GO), and silicon dioxide (SiO_2_), into PMMA materials has been investigated by various scholars, revealing a general tendency: an initial enhancement in mechanical properties bending strength, bending elastic modulus, and tensile strength of PMMA materials, followed by a decrement (Checinska et al., 2022; Sahm et al., 2023; Ahamad Said et al., 2022; Alzayyat et al., 2022; Wang et al., 2019). Literature elucidates that the mechanism underpinning the nanoparticle-induced enhancement of mechanical strength in PMMA materials involves the induction of stress aggregation by the nanoparticle crystal particles. Upon integration into PMMA materials, these particles promote silver patterns in the surrounding matrix, instigating plastic deformation of the adjacent matrix, thereby absorbing external energy and generating toughness. Concurrently, nanoparticles are also capable of terminating the silver lines, yielding, and imparting a toughening effect. However, an incessant increase in the nano-Ag-ZrP crystal content compromises the dispersion of nanoparticle crystals in PMMA material, initiating agglomeration. Transformed from small to large particulate matter through agglomeration, the nanoparticle crystals reduce in surface area, forming a stress concentration zone, and their adhesion to PMMA material deteriorates. The manifestation of numerous defective structures within the material consequently undermines the mechanical properties of PMMA materials (Daelemans et al., 2016; Zeidi et al., 2023; Wang et al., 2017; He et al., 2018). In the current experiment, SEM observation of the fracture surface of each group of specimens, coupled with analysis post-mechanical properties test, affords a viewpoint aligning with preceding studies.

## 4 Conclusion

The morphological characteristics, functional groups, crystal structures, and elemental composition of commercially available nano-Ag-ZrP were analyzed employing SEM, TEM, FT-IR, XRD, and XPS. The material, initially presumed to be nano-Ag-ZrP, demonstrated optimal antibacterial activity against *S. mutans* and *E. coli* when incorporated at a 3.0%wt into the room-temperature cured PMMA material, achieving antibacterial rates of 98.00% and 92.98%, respectively. A concentration of 2.0%wt resulted in antibacterial rates of 93.38% against *S. mutans* and 75.09% against *E. coli* in the PMMA material. Mechanical property testing revealed that room-temperature-curing PMMA materials exhibited peak mechanical properties when the concentration of nano-Ag-ZrP antibacterial agent was within 1.0%~1.5%wt. When the antimicrobial agent concentration was increased to 2.0%wt, the mechanical strength of the PMMA material did not diminish compared to the 0%wt group, while concentrations starting from 2.5%wt resulted in reduced mechanical strength relative to the 0%wt group. Furthermore, cytotoxicity was not detected at nano-Ag-ZrP concentrations below 3.0%wt, indicating commendable biosafety. Consequently, future research aiming to enhance the antimicrobial and mechanical properties of PMMA materials should prioritize augmenting antimicrobial properties without compromising the mechanical integrity of the PMMA materials. In summary, it is recommended that the incorporation of nano-Ag-ZrP (RHA-1F-II) in room-temperature curing PMMA material be maintained within 1.5%wt ~ 2.0%wt.

## Data availability statement

The raw data supporting the conclusions of this article will be made available by the authors, without undue reservation.

## Ethics statement

The studies involving humans were approved by Stomatology Hospital of Jilin University. The studies were conducted in accordance with the local legislation and institutional requirements. Written informed consent for participation in this study was provided by the participants’ legal guardians/next of kin.

## Author contributions

XC: Writing – original draft, Writing – review & editing. TY: Data curation, Methodology, Supervision, Writing – review & editing. SS: Methodology, Writing – review & editing. AL: Conceptualization, Data curation, Writing – review & editing. XW: Formal Analysis, Funding acquisition, Methodology, Resources, Supervision, Writing – review & editing.
